# Fosfomycin Resistance Dynamics in Major Uropathogens: A 2013–2025 Integrated Disease Surveillance of Multidrug-Resistant, Extended-Spectrum Beta-Lactamase-Producing, Non-Extended-Spectrum Beta-Lactamase, and Enterococcal Urinary Isolates

**DOI:** 10.3390/pathogens15070758

**Published:** 2026-07-20

**Authors:** Umar Saeed, Rizwan Uppal, Gohar Zaman, Muhammad Rehan Uppal, Zsolt Jenő Szepesváry, Aftab Ahmad Khan, Muhammad Usman Qamar, Zuhaib Ali, Zahra Zahid Piracha

**Affiliations:** 1 Clinical and Biomedical Research Center (CBRC), Foundation University School of Health Sciences (FUSH), Foundation University Islamabad (FUI), Islamabad 44000, Pakistan; 2 Korea University College of Health Sciences, Korea University, Seongbuk-gu, Seoul 02841, Republic of Korea; 3 Faculty of Health, Széchenyi István University, 9026 Győr, Hungary; szepesvary.zsolt.jeno@ga.sze.hu; 4 International Center of Medical Sciences Research (ICMSR), Islamabad 44000, Pakistan; rizwanuppal@idc.net.pk (R.U.); rehanuppal@idc.net.pk (M.R.U.); piracha.zahra@gmail.com (Z.Z.P.); 5 Islamabad Diagnostic Center (IDC), Islamabad 44000, Pakistan; gauharzaman@gmail.com (G.Z.); aftabahmad@idc.net.pk (A.A.K.); zuhaibali@idc.net.pk (Z.A.); 6 Institute of Microbiology, Faculty of Life Sciences, Government College University Faisalabad, Faisalabad 38000, Pakistan; musmanqamar@gcuf.edu.pk; 7 International Center of Medical Sciences Research (ICMSR), Romford RM6 6AX, UK; 8 International Center of Medical Sciences Research (ICMSR), Austin, TX 77418, USA

**Keywords:** fosfomycin resistance, urinary tract infections, *Escherichia coli*, antimicrobial stewardship, *Klebsiella* spp., *Enterococcus* spp., Pakistan

## Abstract

Urinary tract infections are among the most common bacterial infections encountered in clinical practice, with *Escherichia coli* representing the dominant urinary pathogen. Increasing detection of multidrug-resistant and extended-spectrum beta-lactamase (ESBL)-producing uropathogens has narrowed empirical treatment options and renewed interest in fosfomycin. However, local long-term surveillance data on fosfomycin susceptibility remain limited in Pakistan. This study evaluated temporal changes in major urinary isolate categories and fosfomycin susceptibility patterns within a diagnostic laboratory network in Pakistan from 2013 to 2025. An exploratory molecular sub-analysis was also performed to assess selected resistance-associated transcript patterns in archived fosfomycin-susceptible and fosfomycin-resistant isolates. A retrospective laboratory-based, isolate-level analysis was conducted using anonymized urine culture records. The source database included 34,230 urine sample records, from which eligible culture-positive urinary isolates with required organism classification and fosfomycin susceptibility data were included in the final analytical dataset. Analyses were performed across predefined mutually exclusive study intervals. Organism categories included non-ESBL *E. coli*, ESBL-producing *E. coli*, laboratory-coded ESBL *E. coli* 24 variant, *Klebsiella* spp., and *Enterococcus* spp. The ESBL *E. coli* 24 variant was treated as a laboratory reporting category, not as a genomically confirmed clone or sequence type. Fosfomycin resistance was evaluated using interval-based comparisons and odds ratios. A selected subset of 24 archived isolates, including fosfomycin-susceptible and fosfomycin-resistant *E. coli* and *Klebsiella pneumoniae*, was analyzed by RT-qPCR for glpT, uhpT, murA, fosA, fosA3, and blaCTX-M transcript abundance. The final isolate-level analytical dataset included 17,978 eligible urinary isolates. Among urine records with available sex data, female-associated records represented the majority throughout the study period, but this finding reflects laboratory record distribution rather than patient-level UTI prevalence. *E. coli* remained the predominant urinary isolate category. Non-ESBL *E. coli* declined across study intervals, whereas ESBL-associated *E. coli* categories represented a larger proportion of isolates in later years. The laboratory-coded ESBL *E. coli* 24 variant increased in later intervals, although this finding requires cautious interpretation because confirmatory molecular typing was not performed. Fosfomycin resistance showed a non-linear temporal pattern: resistance decreased from the early to the middle interval and then increased markedly to 23.8% during 2021–2025, while susceptibility declined to 60.6% in the same interval. Compared with the middle interval, isolates from 2021–2025 had higher odds of fosfomycin resistance (OR = 3.64, 95% CI: 3.23–4.12; *p* < 0.001). In the exploratory molecular subset, resistant isolates showed lower transcript abundance of selected uptake-associated genes, particularly glpT and uhpT, and higher expression of selected fosfomycin- and ESBL-associated genes, including fosA, fosA3, and blaCTX-M. These findings represent transcriptional associations in selected isolates and do not establish definitive resistance mechanisms. Urinary isolates in this diagnostic-network dataset showed a temporal shift toward greater representation of laboratory-reported ESBL-associated *E. coli* categories and a marked increase in fosfomycin resistance during 2021–2025. The findings support continued local surveillance of urinary pathogens and periodic reassessment of fosfomycin susceptibility for antimicrobial-stewardship guidance. The molecular findings should be interpreted as exploratory transcriptional observations because they were based on a small selected isolate subset and were not supported by genomic, mutational, uptake, or functional validation.

## 1. Introduction

Antimicrobial resistance (AMR) is a major global public-health challenge that compromises the effectiveness of antimicrobial therapy and contributes to prolonged illness, extended hospitalization, increased healthcare expenditure, and higher mortality [1]. Its impact is particularly important in urinary tract infections (UTIs), which are among the most frequently encountered bacterial infections in routine clinical practice. *Escherichia coli* is the predominant urinary pathogen, whereas *Klebsiella* spp. and *Enterococcus* spp. also contribute substantially, particularly in recurrent, complicated, catheter-associated, and healthcare-associated urinary infections. The increasing occurrence of multidrug-resistant (MDR) and extended-spectrum beta-lactamase (ESBL)-producing uropathogens has progressively restricted empirical treatment options and reinforced the importance of local antimicrobial-resistance surveillance and evidence-based antimicrobial-stewardship strategies [2].

Fosfomycin is an older phosphonic acid antimicrobial agent that has regained clinical relevance because of its activity against a broad range of urinary pathogens, including selected MDR Gram-negative isolates. It inhibits an early and essential step in bacterial cell-wall synthesis by targeting UDP-N-acetylglucosamine enolpyruvyl transferase, encoded by murA, thereby disrupting peptidoglycan biosynthesis [3,4]. Because its mechanism of action differs from those of many commonly used antimicrobial classes, fosfomycin remains a valuable therapeutic option for uncomplicated UTIs and, in selected clinical settings, for infections caused by resistant urinary pathogens. Nevertheless, increasing reports of fosfomycin resistance among major uropathogens indicate that its activity cannot be assumed to remain stable and should be assessed through continued local and regional surveillance [5].

Fosfomycin resistance is biologically heterogeneous and may develop through several non-mutually exclusive mechanisms. These include reduced intracellular drug uptake resulting from alterations in the glycerol-3-phosphate and hexose-phosphate transport systems, encoded by glpT and uhpT, respectively; changes affecting the target-associated pathway involving murA; and enzymatic drug inactivation mediated by fosfomycin-modifying determinants such as fosA and fosA3 [5,6]. These mechanisms may coexist with broader antimicrobial-resistance backgrounds, including ESBL-associated phenotypes, thereby complicating the interpretation of fosfomycin susceptibility patterns in routine urinary isolates [6]. However, differences in gene-expression levels alone indicate transcriptional associations and cannot establish causality or directly demonstrate altered transporter function, target modification, resistance-gene carriage, or functional resistance without complementary genomic, mutational, uptake, or other functional analyses.

In Pakistan, AMR among uropathogens represents an increasing clinical and public-health concern in the context of high antimicrobial consumption, inconsistent implementation of antimicrobial-stewardship practices, and limited availability of long-term laboratory surveillance data [7,8]. Although fosfomycin is frequently considered a useful treatment option for resistant urinary isolates, published local evidence remains limited and is commonly derived from relatively short surveillance periods, modest sample sizes, or individual institutional settings [9,10]. Consequently, the long-term behavior of fosfomycin susceptibility among major urinary pathogens encountered in routine diagnostic practice remains insufficiently characterized.

Against this background, the present study was conducted as a retrospective, laboratory-based, isolate-level surveillance analysis of urinary isolates processed within the Islamabad Diagnostic Center laboratory network between 2013 and 2025. The primary objectives were to characterize temporal changes in the distribution of major urinary isolate categories and to evaluate changes in fosfomycin susceptibility and resistance within the defined analytical dataset. In addition to conventional categories, including non-ESBL *E. coli*, ESBL-producing *E. coli*, *Klebsiella* spp., and *Enterococcus* spp., the analysis included the locally recorded laboratory category “ESBL *E. coli* 24 variant” [11,12,13,14,15,16,17,18]. Throughout this manuscript, this term refers exclusively to a distinct laboratory information-system reporting category used in the source database for ESBL-associated *E. coli* isolates. In the absence of confirmatory molecular typing, it should not be interpreted as a genomically confirmed clone, sequence type, phylogenetic lineage, or ESBL enzyme subtype.

To complement the longitudinal surveillance analysis, a focused exploratory molecular sub-analysis was performed using a small selected subset of archived fosfomycin-susceptible and fosfomycin-resistant *E. coli* and *Klebsiella pneumoniae* isolates. This component assessed the relative transcript abundance of the uptake-associated genes glpT and uhpT, the target-associated gene murA, the fosfomycin-resistance-associated genes fosA and fosA3, and the ESBL-associated marker blaCTX-M using reverse-transcription quantitative PCR. The molecular analysis was designed to describe resistance-associated transcriptional patterns in selected isolates and was not intended to establish causal mechanisms, demonstrate functional resistance, or represent the entire 2013–2025 surveillance population. Together, the epidemiological and exploratory molecular components provide a long-term overview of fosfomycin susceptibility patterns and a cautious description of selected transcriptional features associated with fosfomycin-resistant urinary isolates.

## 2. Materials and Methods

### 2.1. Study Design, Setting, and Data Source

This retrospective, laboratory-based surveillance study was conducted in collaboration with the International Center of Medical Sciences Research (ICMSR), Islamabad, Pakistan, using urine-culture and antimicrobial-susceptibility records generated within the Islamabad Diagnostic Center laboratory network. The source database included urine samples processed between January 2013 and December 2025. Routinely available demographic and microbiological variables were extracted from the laboratory information system, including year of sample processing, recorded sex, organism identification, laboratory-reported extended-spectrum beta-lactamase (ESBL) classification where available, and fosfomycin susceptibility interpretation. All records were anonymized before data analysis. The study was based exclusively on routinely generated laboratory records and did not involve direct patient contact, clinical examination, treatment allocation, or clinical follow-up. It was therefore designed as a surveillance analysis of submitted urine records and recovered urinary isolates rather than as a patient-level clinical cohort of confirmed symptomatic urinary tract infection.

Clinical variables that may influence antimicrobial resistance, including inpatient or outpatient status, urinary symptoms, catheterization, pregnancy, comorbidities, previous antimicrobial exposure, recent hospitalization, recurrent urinary tract infection, and community- versus healthcare-associated acquisition, were not consistently available across the surveillance period and could not be incorporated into the analysis. Consequently, the dataset could not reliably distinguish symptomatic urinary tract infection from asymptomatic bacteriuria, colonization, or urine-culture overtesting. The source database comprised 34,230 urine sample records. Culture-negative records, contaminated cultures, non-reportable mixed-growth cultures, records lacking an interpretable organism classification, organisms outside the predefined analytical categories, and records lacking the microbiological or fosfomycin-susceptibility information required for the relevant analysis were excluded from the final isolate-level dataset.

Following these exclusions, 17,978 eligible urinary isolates were included in the pathogen-distribution and fosfomycin-susceptibility analyses. These comprised 5081 isolates from 2013–2016, 4723 isolates from 2017–2020, and 8174 isolates from 2021–2025. The remaining 16,252 source records did not satisfy the microbiological, organism-classification, or susceptibility-data requirements of the final isolate-level analysis. Some source records nevertheless contributed to the descriptive analysis of urine submissions according to recorded sex. The derivation of the final analytical population was summarized in a numerical study-flow diagram. For temporal comparison, the surveillance period was divided into three predefined, mutually exclusive intervals: 2013–2016, 2017–2020, and 2021–2025. These intervals were applied consistently to descriptive analyses, organism-distribution comparisons, fosfomycin-susceptibility summaries, and interval-specific odds-ratio calculations. No calendar year was included in more than one interval. Broader pre-2020 and post-2020 comparisons, where mentioned, were treated only as supplementary descriptive summaries and did not replace the primary three-interval analytical framework. Ethical approval was obtained from the Ethical Review Committee of Islamabad Diagnostic Center under approval number 20200110ERC. The requirement for informed consent was waived because the study involved retrospective analysis of anonymized laboratory records.

### 2.2. Eligibility Criteria and Analytical Framework

The primary analytical unit of the surveillance component was the urinary isolate rather than the individual patient. Findings were therefore reported as distributions of submitted urine records or eligible urinary isolates according to the denominator relevant to each analysis. The findings were not interpreted as unique-patient incidence, population prevalence, or patient-level clinical risk. For the organism-distribution analysis, eligible culture-positive isolates were classified according to the final organism category recorded in the laboratory information system. The principal analytical categories were non-ESBL *Escherichia coli*, ESBL-producing *E. coli*, laboratory-coded ESBL *E. coli* 24 variant, *Klebsiella* spp., and *Enterococcus* spp. Where required for denominator completeness, less frequently reported organisms were retained within a residual “other reportable urinary isolates” category. Records without an interpretable final organism classification were excluded.

The term “ESBL *E. coli* 24 variant” refers to a locally used laboratory information-system or reporting category for a subgroup of ESBL-associated *E. coli* isolates. It was evaluated exclusively as a historical laboratory-coded category. The designation should not be interpreted as a genomically confirmed clone, multilocus sequence type, phylogenetic lineage, or specific ESBL enzyme subtype because whole-genome sequencing, multilocus sequence typing, clonal analysis, and ESBL gene-subtype confirmation were not performed for the surveillance population. For the fosfomycin-susceptibility analysis, eligible isolates were required to have both an interpretable organism classification and a recorded fosfomycin susceptibility result. Isolates recorded as susceptible or resistant were included in the primary binary resistance analysis. Intermediate susceptible, intermediate resistant, unavailable, unknown, missing, and otherwise non-interpretable results were summarized separately and excluded from the resistant-versus-susceptible comparison. Complete patient-level deduplication could not be guaranteed across the full 2013–2025 surveillance period because stable longitudinal patient identifiers were not uniformly available. Where sufficient identifiers were present, repeated records from the same individual showing the same organism and susceptibility phenotype within a short interval were reviewed to reduce obvious duplicate counting. Nevertheless, more than one isolate from the same individual may have been included in the final dataset. Resistance proportions were therefore interpreted as isolate-level surveillance estimates rather than unique-patient estimates.

### 2.3. Bacterial Identification, ESBL Classification, and Fosfomycin Susceptibility Testing

Urinary isolates were identified using routine diagnostic microbiology procedures in operation within the participating laboratory network during the relevant study period. These procedures included growth characteristics on standard urine-culture media, colony morphology, Gram staining where applicable, conventional biochemical testing, and automated or mass-spectrometry-based identification when available. During the earlier surveillance years, organism identification relied predominantly on conventional culture and biochemical methods. Matrix-assisted laser desorption/ionization time-of-flight mass spectrometry was introduced during the later surveillance period, within the 2021–2025 analytical interval, and was used selectively for confirmatory identification or isolates requiring additional species-level resolution. It was not applied systematically to every urinary isolate. The precise implementation year and the proportion of isolates examined using MALDI-TOF MS could not be reconstructed reliably from the archived laboratory information-system records.

Because organism-identification procedures changed during the 12-year surveillance period, the analysis focused primarily on broad, routinely reportable uropathogen categories rather than uncommon species-level classifications. Potential effects of changes in identification methodology were considered when interpreting longitudinal differences in organism distribution. ESBL status was assigned using the routine phenotypic screening and confirmatory procedures in effect at the time of testing. These generally involved detection of reduced susceptibility to third-generation cephalosporins followed by phenotypic confirmation or automated laboratory interpretation according to the applicable workflow. Molecular ESBL typing was not performed across the complete surveillance population. Accordingly, ESBL categories represent laboratory-reported phenotypic classifications rather than sequence-confirmed resistance genotypes.

Fosfomycin susceptibility testing was conducted using the routine antimicrobial-susceptibility methods available during the surveillance period. Disk diffusion was the principal method used for routine urinary isolates, whereas minimum inhibitory concentration-based testing was performed selectively when available or considered necessary within the clinical laboratory workflow. Fosfomycin disk-diffusion testing was performed using glucose-6-phosphate-supplemented disks in accordance with accepted susceptibility-testing requirements. MIC-based testing similarly followed the glucose-6-phosphate requirements applicable to fosfomycin testing at the time. However, the historical database did not retain a complete harmonized field identifying the susceptibility-testing method used for every isolate. Consequently, the proportions of isolates assessed by disk diffusion and MIC-based methods could not be reconstructed reliably according to organism or study interval. The precise MIC platform or reference method used in every historical case was also unavailable. Raw inhibition-zone diameters, individual MIC measurements, reagent lot information, and complete year-specific method documentation were not uniformly retained. The present analysis therefore used the final categorical susceptibility interpretation recorded in the laboratory database. Fosfomycin susceptibility was interpreted according to the Clinical and Laboratory Standards Institute criteria used by the laboratory during the respective testing period. The laboratory generally applied the contemporaneous CLSI M100 standards available at the time of testing. However, an exact year-by-year mapping of the individual CLSI editions and breakpoint tables used from 2013 through 2025 could not be reconstructed from the archived records. This limitation was considered when interpreting temporal susceptibility patterns. Because CLSI interpretive support for fosfomycin is strongest for urinary *E. coli*, fosfomycin results recorded for non-*E. coli* organisms, particularly *Klebsiella* spp., were interpreted cautiously as laboratory-reported surveillance categories rather than definitive organism-specific therapeutic susceptibility classifications. Fosfomycin breakpoint availability and method performance may differ between organisms and testing standards. Susceptibility outcomes were classified according to the categories retained in the historical laboratory database: susceptible, resistant, intermediate susceptible, intermediate resistant, and unavailable or unknown. The intermediate susceptible and intermediate resistant categories represented historical internal reporting codes rather than standardized categories used for primary statistical inference. They were therefore reported descriptively and were not pooled with resistant isolates in the primary binary resistance analysis.

### 2.4. Molecular Characterization of Selected Archived Isolates

#### 2.4.1. Selection of the Molecular Isolate Subset

An exploratory molecular sub-analysis was conducted using 24 archived urinary isolates selected from the laboratory culture collection. The molecular subset comprised four balanced organism–phenotype groups:

Fosfomycin-susceptible *E. coli* (Ec_S, n = 6); Fosfomycin-resistant *E. coli* (Ec_R, n = 6); Fosfomycin-susceptible *Klebsiella pneumoniae* (Kp_S, n = 6); and Fosfomycin-resistant *K. pneumoniae* (Kp_R, n = 6).

Isolates were selected according to confirmed species identification, the fosfomycin susceptibility phenotype recorded in the laboratory database, successful recovery after storage, availability of sufficient viable culture material, and suitability for RNA extraction. Duplicate isolates from the same patient were avoided where adequate identifiers were available. The molecular subset was intentionally balanced according to organism and susceptibility phenotype but was not randomly sampled from the complete surveillance population. It was not designed to represent all 17,978 isolates included in the longitudinal analysis and was not powered for population-wide extrapolation.

Complete isolate-level collection dates were not retained in the de-identified molecular-analysis worksheet in a form that permitted reliable reconstruction of the distribution of all 24 isolates across individual surveillance years. Consequently, the molecular subset should be interpreted as a cross-sectional, phenotype-based sample rather than a temporally structured representation of the 2013–2025 surveillance population.

For analyses focused specifically on ESBL-associated *E. coli*, isolates were further classified according to the phenotypic ESBL status recorded in the laboratory information system. Because this stratification reduced the number of independent biological isolates within individual subgroups, all ESBL-stratified molecular comparisons were considered exploratory. The relevant biological sample sizes were reported in the corresponding Results text and figure legends.

#### 2.4.2. Storage, Recovery, and Standardization of Bacterial Cultures

Archived isolates were maintained at −80 °C in tryptic soy broth containing 20% glycerol. For recovery, a small quantity of frozen culture stock was streaked onto Mueller–Hinton agar and incubated aerobically at 37 °C for 18–24 h. A morphologically consistent colony was subsequently subcultured onto fresh Mueller–Hinton agar to confirm culture purity. Species identification and the recorded fosfomycin susceptibility phenotype were reviewed using the routine identification and susceptibility procedures available in the laboratory before inclusion in the molecular analysis. For RNA preparation, a single purified colony was inoculated into Mueller–Hinton broth and incubated aerobically at 37 °C with orbital agitation until mid-logarithmic growth was reached, corresponding to an optical density at 600 nm of approximately 0.5–0.6. Comparable culture medium, inoculum conditions, incubation temperature, agitation, and growth density were maintained across susceptible and resistant groups to minimize culture-dependent transcriptional variation. The isolates were not exposed to fosfomycin immediately before RNA extraction. The RT-qPCR measurements therefore represent basal transcript abundance under standardized in vitro culture conditions rather than fosfomycin-induced transcription.

#### 2.4.3. Total RNA Extraction and DNase Treatment

Actively growing bacterial cultures were stabilized before RNA extraction using RNAprotect Bacteria Reagent according to the manufacturer’s recommended bacterial RNA-stabilization procedure. Briefly, 1 mL of bacterial culture was mixed with 2 mL of RNAprotect Bacteria Reagent, incubated at room temperature for 5 min, and centrifuged at approximately 5000× *g* for 10 min. The supernatant was removed completely, and the bacterial pellet was processed immediately. Total bacterial RNA was extracted using the RNeasy Mini Kit (QIAGEN, Hilden, Germany; catalogue no. 74104). Bacterial pellets were resuspended in Tris–EDTA buffer containing lysozyme and incubated at room temperature for approximately 5 min to facilitate cell-wall disruption. Guanidinium-containing lysis buffer was subsequently added, and the lysate was homogenized before ethanol-assisted binding to the silica membrane of the RNA-extraction column.

Residual genomic DNA was removed by on-column digestion using the RNase-Free DNase Set (QIAGEN; catalogue no. 79254). DNase I treatment was performed at room temperature for 15 min, after which the spin columns were washed according to the manufacturer’s instructions. Purified RNA was eluted in 30–50 µL of RNase-free water. RNA concentration and purity were assessed using a NanoDrop One spectrophotometer (Thermo Fisher Scientific, Waltham, MA, USA). RNA preparations with an A260/A280 ratio of approximately 1.8–2.1 and sufficient concentration for reverse transcription were used for downstream analysis. RNA samples were maintained on ice during handling and stored at −80 °C until complementary DNA synthesis. No-reverse-transcriptase controls were included to evaluate residual genomic DNA contamination.

#### 2.4.4. Complementary DNA Synthesis

Complementary DNA was synthesized using the RevertAid First Strand cDNA Synthesis Kit (Thermo Fisher Scientific; catalogue no. K1622). For each reaction, 1 µg of purified total RNA was reverse-transcribed in a final reaction volume of 20 µL using random hexamer primers. The reverse-transcription mixture contained purified RNA, random hexamer primers, reaction buffer, RiboLock RNase inhibitor, deoxynucleotide triphosphates, and RevertAid reverse transcriptase. The reactions were incubated at 25 °C for 5 min to facilitate primer annealing, followed by reverse transcription at 42 °C for 60 min and enzyme inactivation at 70 °C for 5 min. Parallel no-reverse-transcriptase controls were prepared without reverse transcriptase. Synthesized cDNA was diluted 1:10 using nuclease-free water before quantitative PCR and was stored at −20 °C until analysis.

#### 2.4.5. Reverse-Transcription Quantitative PCR

Reverse-transcription quantitative PCR was performed using PowerUp SYBR Green Master Mix (Thermo Fisher Scientific Massachusetts, USA; catalogue no. A25742) on a QuantStudio 5 Real-Time PCR System (Applied Biosystems, Foster City, CA, USA). Each reaction was performed in a final volume of 20 µL containing 10 µL of 2× PowerUp SYBR Green Master Mix, forward and reverse primers at a final concentration of 0.4 µM each, 2 µL of diluted cDNA, and nuclease-free water.

The thermal-cycling program consisted of uracil-DNA glycosylase activation at 50 °C for 2 min, polymerase activation and initial denaturation at 95 °C for 2 min, followed by 40 cycles of denaturation at 95 °C for 15 s and combined annealing and extension at 60 °C for 60 s. A post-amplification melting-curve analysis was performed from 60 °C to 95 °C to evaluate product specificity and identify prominent nonspecific products or primer-dimer formation. The analyzed targets were the fosfomycin uptake-associated genes glpT and uhpT, the target-associated gene murA, the fosfomycin-resistance-associated genes fosA and fosA3, and the ESBL-associated marker blaCTX-M. The blaCTX-M assay was interpreted as a broad ESBL-associated transcriptional assay rather than as identification of a specific CTX-M subtype. The 16S ribosomal RNA gene was used as the internal reference for normalization. Each independent clinical isolate represented one biological replicate. RT-qPCR reactions were performed in technical triplicate, and the mean cycle-threshold value of the three technical reactions was used for analysis. Technical replicates showing inconsistent amplification or a cycle-threshold standard deviation greater than 0.5 cycles were repeated.

No-template controls were included for each gene assay to evaluate reagent contamination and nonspecific amplification. No-reverse-transcriptase controls were used to assess residual genomic DNA contamination. Relative transcript abundance was calculated using the 2^−ΔΔCt^ method. For within-species comparisons, the susceptible group of the same species served as the calibrator. Resistant *E. coli* isolates were therefore evaluated relative to susceptible *E. coli*, whereas resistant *K. pneumoniae* isolates were evaluated relative to susceptible *K. pneumoniae*. Cross-species comparisons were considered exploratory because baseline transcript abundance, amplification efficiency, genomic context, and reference-gene behavior may differ between *E. coli* and *K. pneumoniae*. Fold-change values were used for graphical presentation and biological interpretation, whereas statistical inference was performed using ΔCt values. The molecular analysis assessed relative transcript abundance only. It did not directly evaluate resistance-gene carriage, gene copy number, mutations in glpT, uhpT, or murA, plasmid localization, clonal relatedness, transporter activity, intracellular fosfomycin uptake, MurA enzymatic activity, or functional fosfomycin inactivation. The molecular findings were therefore interpreted as resistance-associated transcriptional patterns rather than definitive causal mechanisms.

### 2.5. Study Outcomes

The primary outcomes of the surveillance component were:

The temporal distribution of major urinary isolate categories across the predefined, mutually exclusive study intervals. The temporal distribution of laboratory-reported fosfomycin susceptibility and resistance among eligible urinary isolates.

Secondary outcomes included the distribution of submitted urine records according to recorded sex and the exploratory comparison of resistance-associated transcript abundance between selected fosfomycin-susceptible and fosfomycin-resistant *E. coli* and *K. pneumoniae* isolates.

Because the study was based on retrospective laboratory data, all outcomes were interpreted as laboratory-surveillance measures rather than direct estimates of symptomatic urinary tract infection incidence, patient-level risk, treatment failure, therapeutic response, or mortality.

### 2.6. Statistical Analysis

Statistical analyses of the surveillance dataset were performed using IBM SPSS Statistics, version 26.0 (IBM Corp., Armonk, NY, USA). Molecular-expression analyses and graphical preparation were conducted using GraphPad Prism, version 9.5.1 (GraphPad Software, San Diego, CA, USA). Analyses were conducted primarily at the urinary-isolate level. Categorical variables were summarized as frequencies and percentages. Molecular-expression data were summarized as mean ± standard deviation. Separate denominators were reported for the sex-distribution, organism-distribution, and fosfomycin-susceptibility analyses because record completeness and eligibility differed between these analytical components.

Differences in categorical distributions across surveillance intervals were evaluated using Pearson’s chi-square test. Fisher’s exact test was used where expected cell frequencies were insufficient for valid chi-square approximation. A chi-square test for trend was applied only when an ordered directional hypothesis was appropriate. Because fosfomycin resistance decreased from 2013–2016 to 2017–2020 and subsequently increased during 2021–2025, the temporal pattern was not assumed to be linear. The primary interpretation therefore relied on interval-specific resistance proportions and direct comparisons between predefined study periods rather than on a single constant annual trend estimate.

For the primary fosfomycin-resistance analysis, susceptibility was analyzed as a binary outcome, with resistant isolates compared against susceptible isolates. Isolates categorized as intermediate susceptible, intermediate resistant, unavailable, unknown, missing, or otherwise non-interpretable were excluded from the binary analysis and summarized separately.

Unadjusted odds ratios and corresponding 95% confidence intervals were calculated to compare the odds of fosfomycin resistance between surveillance intervals. The principal comparison evaluated resistance during 2021–2025 relative to 2017–2020. The comparison between 2017–2020 and 2013–2016 was also performed to characterize the reduction in resistance observed during the middle interval.

A multivariable logistic-regression model was not included in the final analysis because sufficiently complete and harmonized covariate information was not consistently available across the complete surveillance period. Accordingly, all odds ratios reported in the Results represent unadjusted interval-based estimates. Recorded sex, organism category, ESBL status, inpatient or outpatient status, catheterization, comorbidities, previous antimicrobial exposure, recent hospitalization, and community- versus healthcare-associated acquisition were not incorporated into an adjusted model.

For RT-qPCR analysis, each independent clinical isolate was treated as one biological replicate. Technical triplicates were averaged before inferential statistical testing. Fold-change values calculated using the 2^−ΔΔCt^ method were used for graphical presentation, whereas statistical analyses were performed using ΔCt values because these are more appropriate for inference on the logarithmic amplification scale. The distribution of ΔCt values was assessed separately for each gene using the Shapiro–Wilk test and inspection of quantile–quantile plots. Homogeneity of variance was assessed using Levene’s test.

For comparisons between two independent groups, a two-sided unpaired Student’s *t*-test was used when assumptions of normality and equal variance were satisfied. Welch’s unpaired *t*-test was used when values were approximately normally distributed but group variances were unequal. For analyses involving three or more independent groups, one-way analysis of variance was used when parametric assumptions were satisfied. Significant omnibus analyses were followed by Tukey’s multiple-comparison test, and Tukey-adjusted *p*-values were used for predefined pairwise comparisons within each gene analysis. Where assumptions for parametric analysis were not satisfied, the Mann–Whitney U test was used for comparisons between two groups, whereas the Kruskal–Wallis test followed by Dunn’s multiple-comparison procedure was used for analyses involving more than two groups. Adjustment for multiple pairwise comparisons was applied within each individual gene analysis using Tukey’s or Dunn’s procedure, as appropriate. No additional global false-discovery-rate correction was applied across the six separately assessed genes because the molecular component was predefined as exploratory. Marginally significant findings were therefore interpreted cautiously. All statistical tests were two-sided, and *p* < 0.05 was considered statistically significant. Exact *p*-values were reported where informative, whereas values below 0.001 were presented as *p* < 0.001. All findings were interpreted in the context of the retrospective isolate-level design, incomplete patient-level deduplication, limited clinical metadata, possible historical changes in organism-identification and susceptibility-testing procedures, absence of exact year-by-year CLSI edition mapping, and the small selected nature of the 24-isolate molecular subset.

## 3. Results

A total of 34,230 urine sample records were identified in the retrospective laboratory database for the period from January 2013 through December 2025. After exclusion of culture-negative records, contaminated or non-reportable mixed-growth cultures, records lacking interpretable organism-level classification, organisms outside the predefined analytical categories, and records without the microbiological or fosfomycin-susceptibility information required for the relevant analysis, 17,978 eligible urinary isolates were included in the final isolate-level analytical dataset.

The final analytical population comprised 5081 isolates from 2013–2016, 4723 isolates from 2017–2020, and 8174 isolates from 2021–2025. The difference between the 34,230 source records and the 17,978 eligible isolates reflects the fact that not every submitted urine sample yielded a culture-positive, reportable urinary isolate with the organism-classification and susceptibility information required for inclusion.

Because data completeness differed across analytical components, the denominators used for the recorded-sex distribution, organism distribution, and fosfomycin-susceptibility analyses were not identical. The denominator relevant to each analysis is therefore reported separately. All findings represent sample- or isolate-level surveillance observations and should not be interpreted as patient-level estimates of symptomatic urinary tract infection incidence or prevalence.

### 3.1. Distribution of Urine Records According to Recorded Sex

**Figure 1:**
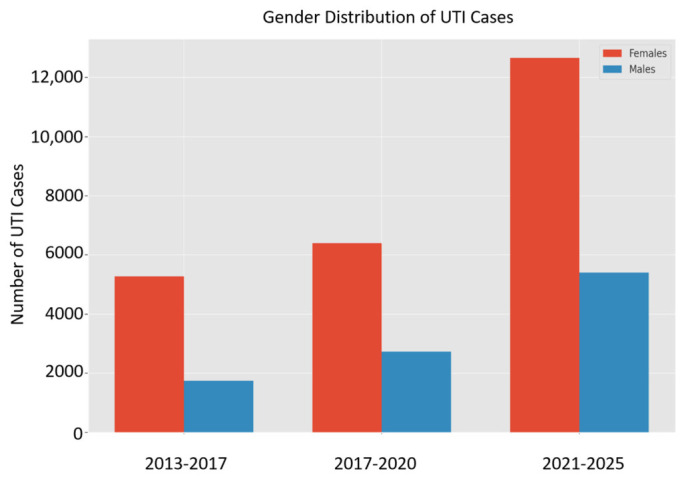
Distribution of urine sample records according to recorded sex across the study intervals. Bars show the percentages of records associated with females and males within each interval. Denominators were 7028 records for 2013–2016, 9136 records for 2017–2020, and 18,064 records for 2021–2025. The figure represents the distribution of submitted laboratory records and not unique-patient UTI incidence.

Recorded sex was available for 34,228 of the 34,230 urine sample records. Records with female sex recorded constituted the majority of urine submissions throughout the surveillance period. During 2013–2016, 5280 of 7028 records were associated with females (75.1%), whereas 1748 were associated with males (24.9%). During 2017–2020, 6402 of 9136 records were associated with females (70.1%) and 2734 with males (29.9%). During 2021–2025, 12,664 of 18,064 records were associated with females (70.1%) and 5400 with males (29.9%) as shown in Figure 1.

Although the absolute number of submitted records increased substantially over time, the proportional distribution according to recorded sex remained stable between the middle and most recent intervals. The proportion of male-associated records increased from 24.9% in 2013–2016 to 29.9% in 2017–2020 and remained 29.9% during 2021–2025. The distribution of recorded sex differed significantly across the three intervals according to Pearson’s chi-square test (*p* < 0.001). However, because this analysis was based on submitted urine records rather than unique patients with clinically confirmed symptomatic infection, the finding reflects the sex distribution of laboratory submissions and should not be interpreted as sex-specific UTI prevalence.

### 3.2. Distribution of Major Uropathogen Categories

Among the 17,978 eligible urinary isolates included in the organism-distribution analysis, *Escherichia coli* remained the predominant organism category across the surveillance period. However, the relative composition of *E. coli* subcategories changed over time, with a progressive decline in non-ESBL *E. coli* and a greater proportional contribution of ESBL-associated *E. coli* categories during the later intervals.

**Figure 2:**
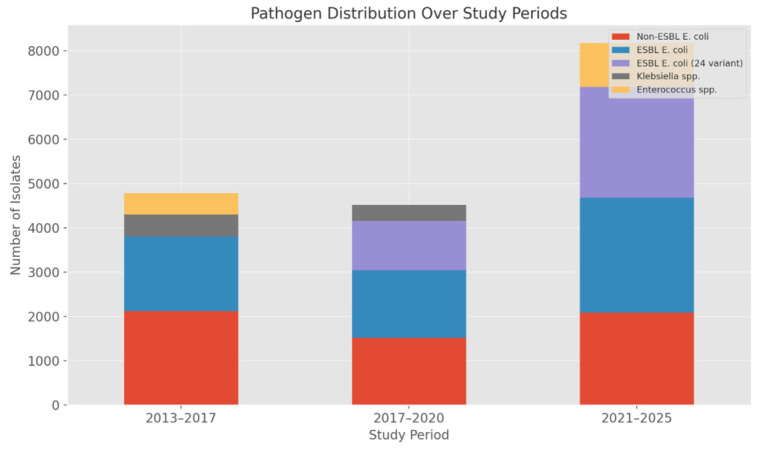
Distribution of major urinary isolate categories across the study intervals. Data are presented as percentages of eligible urinary isolates within each interval. Denominators were 5081 isolates for 2013–2016, 4723 isolates for 2017–2020, and 8174 isolates for 2021–2025. The ESBL *E. coli* 24 variant represents a laboratory-coded ESBL-associated subgroup and not a genomically confirmed clone or sequence type.

In 2013–2016 (n = 5081), non-ESBL *E. coli* was the most frequently recorded category, accounting for 2122 isolates (41.7%) as shown in Figure 2. ESBL-producing *E. coli* accounted for 1679 isolates (33.0%), followed by *Klebsiella* spp. with 502 isolates (9.9%) and *Enterococcus* spp. with 482 isolates (9.5%). The remaining 296 isolates (5.8%) were classified within other reportable urinary isolate categories.

In 2017–2020 (n = 4723), non-ESBL *E. coli* decreased to 1522 isolates (32.2%), while ESBL-producing *E. coli* accounted for 1525 isolates (32.3%). The laboratory-coded ESBL *E. coli* 24 variant category was recorded in 1108 isolates (23.5%), and *Klebsiella* spp. accounted for 368 isolates (7.8%). The remaining 200 isolates (4.2%) were assigned to other reportable urinary isolate categories.

In 2021–2025 (n = 8174), non-ESBL *E. coli* declined further to 2088 isolates (25.5%). ESBL-producing *E. coli* accounted for 2598 isolates (31.8%), while the laboratory-coded ESBL *E. coli* 24 variant category increased to 2499 isolates (30.6%). *Enterococcus* spp. accounted for 989 isolates (12.1%). The categories reported for this interval accounted for the complete analytical denominator.

The designation “ESBL *E. coli* 24 variant” represents a historical laboratory information-system category for an ESBL-associated *E. coli* subgroup. It should not be interpreted as a genomically confirmed clone, sequence type, phylogenetic lineage, or ESBL enzyme subtype because confirmatory molecular typing was not performed for the surveillance population.

The overall distribution of organism categories differed significantly across the three intervals (*p* < 0.001). The principal descriptive pattern was a reduction in the proportion of non-ESBL *E. coli* together with increased representation of ESBL-associated *E. coli* categories during the later surveillance periods. Because interval denominators differed, proportional distributions provide a more appropriate basis for comparison than absolute isolate counts alone.

### 3.3. Fosfomycin Susceptibility Distribution

**Figure 3:**
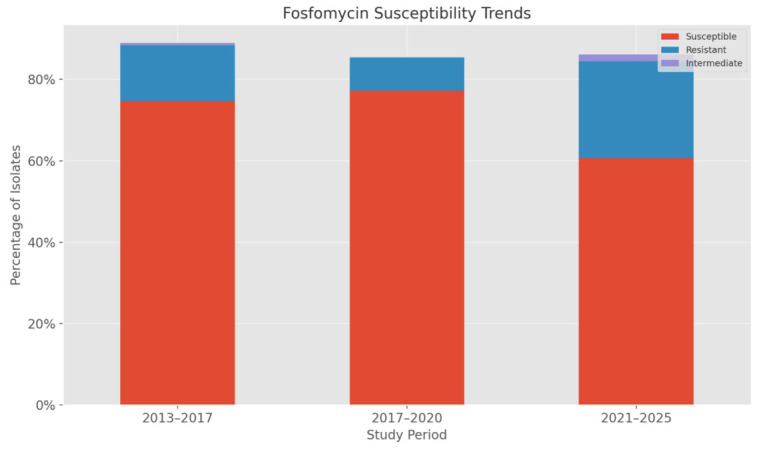
Fosfomycin susceptibility distribution across the study intervals. Bars show the percentages of eligible urinary isolates classified as susceptible, resistant, intermediate susceptible, intermediate resistant, or unavailable/unknown. Denominators were 5081 isolates for 2013–2016, 4723 isolates for 2017–2020, and 8174 isolates for 2021–2025. Intermediate and unavailable categories were excluded from the primary resistant-versus-susceptible analysis.

The distribution of laboratory-reported fosfomycin susceptibility categories varied significantly across the three analytical intervals. In 2013–2016 (n = 5081), 74.7% of isolates were classified as susceptible and 13.7% as resistant. Intermediate susceptible and intermediate resistant categories were uncommon, accounting for 0.02% and 0.51% of isolates, respectively, as shown in Figure 3. The remaining approximately 11.1% had unavailable, unknown, missing, or otherwise non-interpretable fosfomycin results. In 2017–2020 (n = 4723), the proportion of susceptible isolates increased to 77.2%, whereas resistance decreased to 8.1%. Intermediate susceptible and intermediate resistant categories accounted for 0.13% and 0.02%, respectively. Approximately 14.6% of records had unavailable, unknown, missing, or non-interpretable fosfomycin results.

In 2021–2025 (n = 8174), the proportion of susceptible isolates declined to 60.6%, while resistance increased to 23.8%. The intermediate susceptible category increased to 1.7%, whereas intermediate resistant results remained uncommon at 0.02%. Approximately 13.9% of records had unavailable, unknown, missing, or non-interpretable results. Intermediate susceptible, intermediate resistant, unavailable, unknown, and missing results were described separately and were not combined with resistant isolates in the primary binary resistance analysis. The overall distribution of fosfomycin susceptibility categories differed significantly across the intervals (*p* < 0.001). The resistance pattern was non-monotonic: resistance decreased between 2013–2016 and 2017–2020 and subsequently increased markedly during 2021–2025. The temporal change is therefore more accurately characterized as a decline followed by a substantial increase rather than as a constant year-by-year rise.

### 3.4. Interval-Based Risk Analysis of Fosfomycin Resistance

Unadjusted interval-based odds-ratio analyses were performed using fosfomycin-resistant isolates as the outcome and susceptible isolates as the reference category. Isolates recorded as intermediate susceptible, intermediate resistant, unavailable, unknown, missing, or otherwise non-interpretable were excluded from these binary comparisons. Compared with isolates from 2013–2016, isolates recovered during 2017–2020 had lower odds of fosfomycin resistance (unadjusted OR = 0.58, 95% CI: 0.49–0.68; *p* < 0.001). In contrast, isolates from 2021–2025 had substantially higher odds of fosfomycin resistance than isolates from 2017–2020 (unadjusted OR = 3.64, 95% CI: 3.23–4.12; *p* < 0.001). These comparisons indicate that the principal deterioration in fosfomycin susceptibility occurred during the most recent surveillance interval. Because the interval-specific pattern consisted of an initial decline followed by a marked increase, no constant linear annual increase was inferred from the data. No adjusted multivariable regression estimates are presented.

### 3.5. Exploratory Molecular Analysis of Selected Fosfomycin-Susceptible and Fosfomycin-Resistant Isolates

RT-qPCR was performed on a selected exploratory subset of 24 archived urinary isolates. The subset comprised fosfomycin-susceptible *E. coli* (Ec_S, n = 6), fosfomycin-resistant *E. coli* (Ec_R, n = 6), fosfomycin-susceptible *Klebsiella pneumoniae* (Kp_S, n = 6), and fosfomycin-resistant *K. pneumoniae* (Kp_R, n = 6). Each biological replicate represented an independent clinical isolate, and technical replicate Ct values were averaged before analysis. Relative expression values are presented as 2^−ΔΔCt^ fold changes, whereas statistical inference was performed using ΔCt values, as described in the Methods. Because the molecular subset was small, deliberately selected, and not temporally structured across the full surveillance period, the findings should be interpreted as exploratory transcriptional associations rather than mechanistic proof or population-level estimates applicable to the complete 17,978-isolate dataset.

#### Expression of Transporter- and Target-Associated Genes

**Figure 4:**
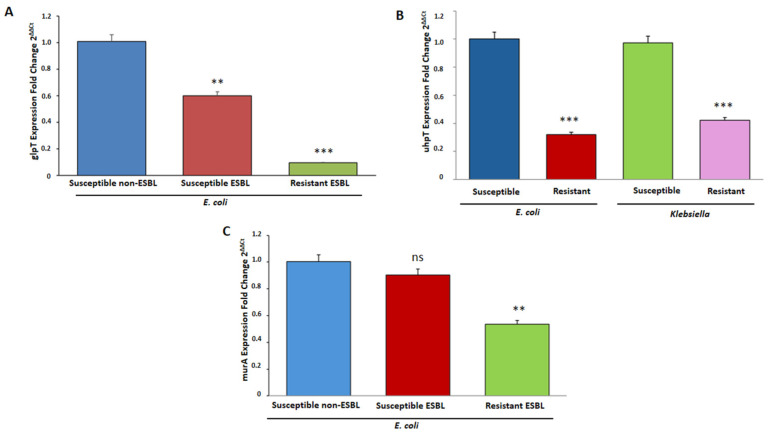
Relative expression of transporter- and target-associated genes in selected urinary isolates. RT-qPCR was used to assess the relative transcript abundance of glpT (**A**), uhpT (**B**), and murA (**C**). Values are presented as mean ± SD of independent biological isolates. Technical replicate Ct values were averaged before analysis. Panels (**A**,**C**) show ESBL-stratified *E. coli* subgroups, whereas Panel (**B**) shows susceptible and resistant *E. coli* and *K. pneumoniae* groups. Fold-change values were calculated using the 2^−ΔΔCt^ method, while statistical inference was performed using ΔCt values. Pairwise comparisons and multiplicity adjustment were performed as described in the Methods. *p* values < 0.01 (**); <0.001 (***) were considered statistically significant.

Expression of glpT was evaluated in ESBL-stratified *E. coli* subgroups (Figure 4A). Susceptible non-ESBL *E. coli* showed a mean relative expression of 1.01 ± 0.15, whereas susceptible ESBL *E. coli* showed lower expression of 0.60 ± 0.07. Resistant ESBL *E. coli* showed the lowest expression, at 0.09 ± 0.02. Pairwise analysis of ΔCt values demonstrated significantly lower glpT transcript abundance in susceptible ESBL *E. coli* than in susceptible non-ESBL *E. coli* (*p* < 0.001). Expression was also significantly lower in resistant ESBL *E. coli* than in susceptible non-ESBL *E. coli* (*p* < 0.001) and susceptible ESBL *E. coli* (*p* < 0.001). These findings demonstrate an association between reduced glpT transcript abundance and the resistant ESBL *E. coli* phenotype in the selected subset.

A similar pattern was observed for uhpT (Figure 4B). In *E. coli*, susceptible isolates showed a relative expression of 1.00 ± 0.02, whereas resistant isolates showed reduced expression of 0.32 ± 0.01 (*p* < 0.001). In *K. pneumoniae*, susceptible isolates showed expression of 0.97 ± 0.02, compared with 0.42 ± 0.01 in resistant isolates (*p* < 0.001). The exploratory cross-species comparison also showed lower uhpT expression in resistant *E. coli* than in resistant *K. pneumoniae* (0.32 ± 0.01 versus 0.42 ± 0.01; *p* < 0.001). This cross-species difference should be interpreted cautiously because baseline expression and amplification characteristics may differ between species.

Expression of murA was assessed in ESBL-stratified *E. coli* groups (Figure 4C). Susceptible non-ESBL *E. coli* showed a mean relative expression of 1.00 ± 0.08, while susceptible ESBL *E. coli* showed a modest reduction to 0.90 ± 0.15. This difference was not statistically significant (*p* = 0.134). Resistant ESBL *E. coli* showed lower murA expression of 0.54 ± 0.09 compared with susceptible non-ESBL *E. coli* (*p* < 0.001) and susceptible ESBL *E. coli* (*p* < 0.001). Collectively, the selected resistant isolates showed lower transcript abundance of the uptake-associated genes glpT and uhpT, together with lower murA expression in resistant ESBL *E. coli*. These observations identify transcriptional associations but do not establish impaired fosfomycin uptake, altered transporter activity, target mutation, or functional modification of MurA.

### 3.6. Expression of Fosfomycin- and ESBL-Associated Resistance Genes in Selected Isolates

**Figure 5:**
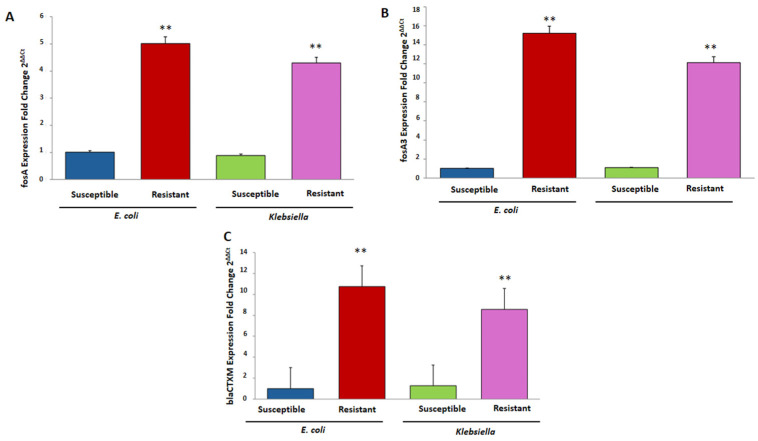
Relative expression of fosfomycin-modifying and ESBL-associated genes in selected urinary isolates. RT-qPCR was used to assess the relative transcript abundance of fosA (**A**), fosA3 (**B**), and blaCTX-M (**C**) in selected fosfomycin-susceptible and fosfomycin-resistant *E. coli* and *K. pneumoniae* isolates. Values are presented as mean ± SD of independent biological isolates. Technical replicate Ct values were averaged before analysis. Fold changes were calculated using the 2^−ΔΔCt^ method, while statistical inference was performed using ΔCt values. Pairwise comparisons and multiplicity adjustment were conducted as described in the Methods. blaCTX-M was interpreted as an ESBL-associated marker and not as a direct fosfomycin-resistance mechanism. *p* < 0.01 (**) was considered statistically significant.

RT-qPCR analysis showed higher fosA expression in fosfomycin-resistant isolates of both species (Figure 5A). In *E. coli*, relative expression increased from 1.01 ± 0.14 in susceptible isolates to 5.01 ± 0.81 in resistant isolates (*p* < 0.001). In *K. pneumoniae*, expression increased from 0.89 ± 0.08 in susceptible isolates to 4.29 ± 0.63 in resistant isolates (*p* < 0.001). The difference between resistant *E. coli* and resistant *K. pneumoniae* was not statistically significant (*p* = 0.118). The corresponding difference between the susceptible groups was also not significant (*p* = 0.100). These findings indicate that increased fosA transcript abundance was associated with the resistant phenotype in both species.

A more pronounced difference was observed for fosA3 expression (Figure 5B). In *E. coli*, relative expression increased from 1.01 ± 0.14 in susceptible isolates to 15.20 ± 2.46 in resistant isolates (*p* < 0.001). In *K. pneumoniae*, expression increased from 1.09 ± 0.10 in susceptible isolates to 12.14 ± 1.79 in resistant isolates (*p* < 0.001). Resistant *E. coli* showed higher fosA3 expression than resistant *K. pneumoniae* (*p* = 0.036), whereas the difference between susceptible groups was not statistically significant (*p* = 0.235). Although fosA3 showed clear separation between the selected susceptible and resistant groups, the study did not evaluate receiver-operating characteristics, sensitivity, specificity, predictive performance, or external validation. It should therefore not be regarded as a validated discriminatory biomarker on the basis of these data.

Expression of the ESBL-associated gene blaCTX-M was also assessed (Figure 5C). In *E. coli*, relative expression increased from 1.01 ± 0.14 in susceptible isolates to 10.75 ± 1.74 in resistant isolates (*p* < 0.001). In *K. pneumoniae*, expression increased from 1.26 ± 0.11 in susceptible isolates to 8.58 ± 1.27 in resistant isolates (*p* < 0.001). Resistant *E. coli* showed higher blaCTX-M expression than resistant *K. pneumoniae* (*p* = 0.036). Among susceptible isolates, *K. pneumoniae* showed modestly higher expression than *E. coli* (*p* = 0.006).

Overall, resistant isolates within the selected molecular subset showed higher transcript abundance of fosA, fosA3, and blaCTX-M. However, blaCTX-M is an ESBL-associated determinant and not a direct fosfomycin-resistance mechanism. Moreover, the analysis did not include comprehensive gene-carriage testing, plasmid characterization, gene copy-number analysis, mutational assessment, whole-genome sequencing, or full isolate-level MDR profiling. These findings should therefore be interpreted as resistance-associated transcriptional observations rather than evidence of coordinated regulation, validated molecular biomarkers, or definitive causal resistance mechanisms.

## 4. Discussion

This retrospective, laboratory-based surveillance study characterized temporal changes in the distribution of major urinary isolates and laboratory-reported fosfomycin susceptibility within a diagnostic laboratory network in Pakistan from 2013 through 2025. The principal findings were a progressive decline in the proportional representation of non-ESBL *Escherichia coli*, continued substantial representation of phenotypically identified ESBL-producing *E. coli*, increased recording of the laboratory-coded ESBL *E. coli* 24 variant category during the later intervals, and a marked increase in fosfomycin resistance during 2021–2025. These findings indicate that the isolate profile captured by the participating laboratory network shifted over time toward greater representation of laboratory-reported ESBL-associated categories and reduced fosfomycin susceptibility. These observations should, however, be interpreted within the context of the study design. The analysis was based on submitted urine records and urinary isolates rather than unique patients with clinically confirmed symptomatic urinary tract infection. It therefore describes laboratory-surveillance patterns and does not provide patient-level estimates of UTI incidence, prevalence, treatment failure, or clinical outcome.

The changing distribution of *E. coli* categories is important because ESBL-associated urinary isolates commonly restrict empirical treatment options and increase reliance on local susceptibility information. In the present dataset, non-ESBL *E. coli* declined from 41.7% during 2013–2016 to 25.5% during 2021–2025. In contrast, laboratory-reported ESBL-associated *E. coli* categories accounted for a larger proportion of isolates during the later surveillance intervals. The conventional ESBL-producing *E. coli* category remained comparatively stable, whereas much of the increase in the overall ESBL-associated representation was attributable to the laboratory-coded ESBL *E. coli* 24 variant category. This distinction is essential. In the present study, “ESBL *E. coli* 24 variant” represents a historical laboratory information-system or reporting category and not a genomically confirmed clone, multilocus sequence type, phylogenetic lineage, or ESBL enzyme subtype.

The increased recording of this category may reflect an actual change in the distribution of ESBL-associated isolates. However, it may also have been influenced by changes in laboratory coding, organism classification, reporting practices, diagnostic workflow, or information-system configuration during the surveillance period. Consequently, the findings do not support describing this category as an emerging clone or genetic variant. Confirmation would require standardized phenotypic classification together with molecular ESBL subtyping, clonal analysis, multilocus sequence typing, or whole-genome sequencing. A second major finding was the non-linear temporal pattern of fosfomycin resistance. Resistance decreased from 13.7% during 2013–2016 to 8.1% during 2017–2020, followed by a marked increase to 23.8% during 2021–2025. The results therefore do not demonstrate a constant year-by-year increase. Instead, they indicate an initial decline followed by substantial deterioration in susceptibility during the most recent interval.

The unadjusted interval-based analysis supported this interpretation. Isolates from 2017–2020 had lower odds of fosfomycin resistance than isolates from 2013–2016, whereas isolates from 2021–2025 had markedly higher odds of resistance than those from 2017–2020. These odds ratios were not adjusted for organism distribution, ESBL status, clinical setting, previous antimicrobial exposure, or other potential confounders because complete and harmonized covariate data were not available across the entire surveillance period. The estimates should therefore be understood as descriptive interval-level associations rather than causal effects of calendar period.

Several factors may have contributed to the observed fluctuation in fosfomycin resistance. These include changes in antimicrobial prescribing, population case mix, referral patterns, culture-submission practices, representation of ESBL-associated organisms, inpatient or outpatient sampling, prior antimicrobial exposure, and healthcare-associated infection burden. Changes in organism-identification procedures, antimicrobial-susceptibility testing methods, interpretive standards, and laboratory reporting rules may also have contributed. Because these factors could not be evaluated consistently, the study cannot determine the reason for the temporary decline during 2017–2020 or the subsequent increase during 2021–2025. The resistance level observed during the most recent interval has relevant antimicrobial-stewardship implications. Fosfomycin is commonly considered an oral treatment option for uncomplicated lower urinary tract infections and, in selected settings, for urinary infections caused by resistant Gram-negative organisms. However, the finding that 23.8% of eligible isolates were recorded as resistant during 2021–2025 indicates that fosfomycin susceptibility should not be assumed within this laboratory population.

Empirical fosfomycin use should therefore be informed by current local antibiograms, organism identity, clinical presentation, infection complexity, and isolate-specific susceptibility testing where available. The present study did not include treatment selection, dose, duration, adherence, microbiological clearance, recurrence, or clinical follow-up. It consequently cannot determine whether the observed laboratory resistance translated into treatment failure or adverse clinical outcomes. Nevertheless, the findings support regular reassessment of fosfomycin activity within local antimicrobial-stewardship programs.

The increased representation of *Enterococcus* spp. during 2021–2025 is also noteworthy. Although *Enterococcus* spp. remained less frequent than *E. coli*, its increased proportional contribution may indicate a broader change in the urinary isolate spectrum received by the diagnostic network. This change could reflect differences in patient mix, catheter-associated sampling, prior antimicrobial exposure, hospitalization, recurrent infection, referral source, long-term-care exposure, or laboratory testing behavior. Because the retrospective database lacked these clinical variables, the reason for the increase could not be determined. The finding nevertheless reinforces the importance of including both Gram-negative and Gram-positive organisms in longitudinal urinary-pathogen surveillance rather than restricting monitoring exclusively to *E. coli*.

The exploratory molecular component identified differences in transcript abundance between selected fosfomycin-susceptible and fosfomycin-resistant isolates. Resistant isolates showed lower expression of the uptake-associated genes glpT and uhpT. Resistant ESBL-associated *E. coli* also showed lower murA transcript abundance. In contrast, resistant *E. coli* and *Klebsiella pneumoniae* showed higher transcript abundance of fosA and fosA3, while blaCTX-M expression was also higher in the selected resistant groups. These findings are biologically compatible with pathways previously associated with fosfomycin resistance and ESBL-associated resistance backgrounds. Reduced expression of glpT and uhpT may be associated with reduced activity of fosfomycin uptake systems, whereas increased expression of fosA or fosA3 may be associated with enhanced enzymatic drug inactivation. However, the present experiments measured relative transcript abundance only.

The study did not directly assess transporter activity, intracellular fosfomycin concentration, fosfomycin uptake, MurA enzymatic function, target-site mutations, resistance-gene copy number, plasmid localization, promoter activity, or protein abundance. Reduced murA expression should therefore not be interpreted as evidence of target mutation or functional target modification. Likewise, higher fosA or fosA3 expression does not independently prove that these genes caused the resistant phenotype. The interpretation of blaCTX-M also requires particular caution. blaCTX-M is an ESBL-associated determinant and not a direct fosfomycin-resistance gene. Its increased expression among selected fosfomycin-resistant isolates may reflect coexistence of fosfomycin resistance within a broader ESBL-associated resistance background. The findings do not demonstrate coordinated regulation between blaCTX-M and the fosfomycin-resistance-associated genes, nor do they establish plasmid co-localization, genetic linkage, or a shared causal mechanism.

The molecular subset was deliberately small and selected. It comprised 24 archived isolates, with six independent isolates in each principal organism–phenotype group. This balanced design permitted exploratory comparison of susceptible and resistant phenotypes but was not powered for population-level inference. The isolates were not randomly sampled from the full surveillance dataset, and complete isolate-level dates were unavailable in the de-identified molecular worksheet. The molecular subset therefore did not constitute a temporally structured sample of the 2013–2025 surveillance population. Consequently, the RT-qPCR findings cannot explain the increase in fosfomycin resistance observed during 2021–2025. They should instead be interpreted as cross-sectional transcriptional differences between selected susceptible and resistant isolates recovered from the archived collection.

Several limitations should be considered when interpreting the surveillance findings. First, the study was retrospective and depended on the completeness, consistency, and accuracy of routine laboratory records. Data collected for diagnostic purposes may contain missing fields, historical coding differences, and changes in reporting conventions that are difficult to harmonize retrospectively.

Second, the primary analytical unit was the isolate rather than the individual patient. Complete patient-level deduplication could not be guaranteed because stable longitudinal identifiers were not consistently available across all years. More than one isolate from the same individual may therefore have contributed to the dataset. This could increase the observed resistance proportion if individuals with recurrent, persistent, complicated, or resistant infections underwent repeated urine cultures.

Third, the absence of clinical metadata substantially limited interpretation. The database did not consistently include inpatient or outpatient status, urinary symptoms, catheterization, pregnancy, comorbidities, previous antimicrobial exposure, recent hospitalization, recurrent UTI, referral source, or community- versus healthcare-associated acquisition. The study could therefore not distinguish symptomatic UTI from asymptomatic bacteriuria, colonization, or potentially unnecessary urine-culture testing.

Fourth, bacterial-identification methods changed during the surveillance period. Earlier identification relied mainly on conventional culture and biochemical procedures, whereas MALDI-TOF MS became available during the later surveillance interval and was used selectively. The precise implementation year and proportion of isolates assessed by MALDI-TOF MS could not be reconstructed. Such changes may have affected organism categorization, particularly for less common species, although the use of broad organism categories reduced some of this uncertainty.

Fifth, antimicrobial-susceptibility testing and interpretation may not have been completely uniform throughout the 12-year period. Disk diffusion was the principal fosfomycin testing method, while MIC-based testing was used selectively. The historical database did not consistently retain the method used for each isolate, raw zone diameters, individual MIC values, or sufficient information to reconstruct the testing-method distribution across organisms and intervals. The laboratory applied contemporaneous CLSI criteria, but the exact year-by-year editions and breakpoint tables could not be mapped retrospectively. Moreover, CLSI interpretive support for fosfomycin is strongest for urinary *E. coli*, and results recorded for non-*E. coli* organisms should therefore be interpreted as laboratory-reported surveillance categories rather than definitive organism-specific therapeutic classifications. These methodological limitations mean that part of the observed temporal variation may reflect changes in testing or interpretation in addition to true biological changes in resistance.

Sixth, the internal intermediate susceptible and intermediate resistant categories reflected historical laboratory information-system codes rather than standardized categories used in the primary binary analysis. These records, together with unavailable, unknown, and missing results, were excluded from resistant-versus-susceptible odds-ratio comparisons. Changes in the proportion of such records over time could have influenced interval-specific denominators.

Seventh, the ESBL *E. coli* 24 variant category was not supported by confirmatory molecular typing. Its apparent increase should therefore remain a descriptive laboratory-surveillance observation. Terms such as “emergence,” “expansion,” or “clonal spread” would be inappropriate without sequencing, standardized subtype confirmation, or clonal analysis.

Eighth, the molecular analysis was restricted to a small selected subset and did not include comprehensive resistance-gene carriage testing, plasmid characterization, mutational analysis, protein-level measurements, direct uptake assays, complementation studies, or whole-genome sequencing. The molecular findings therefore cannot establish causality, coordinated gene regulation, biomarker performance, or mechanisms generalizable to the complete surveillance population.

Finally, the generalizability of the findings is limited. Although the study included a large number of records from an established diagnostic laboratory network, it reflects the population submitting samples to that network. It may not represent rural populations, public-sector institutions, primary-care settings, tertiary-care inpatient populations, long-term-care facilities, or all geographic regions of Pakistan. National estimates should therefore not be inferred directly from these data. Despite these limitations, the study provides long-term laboratory-surveillance evidence of important changes within the analyzed diagnostic network. The proportional contribution of non-ESBL *E. coli* declined, laboratory-reported ESBL-associated categories became more prominent, and fosfomycin resistance reached its highest level during 2021–2025. The findings support continued surveillance of urinary pathogens, regular updating of local antibiograms, cautious empirical use of fosfomycin, and strengthened antimicrobial-stewardship practices. The results should nevertheless be interpreted as surveillance signals rather than evidence of causation, patient-level clinical risk, national prevalence, or definitive molecular mechanisms.

### Future Recommendations

Future surveillance studies should use prospective designs or more comprehensively annotated retrospective databases. Stable patient-level identifiers should be retained to permit deduplication and differentiation between first isolates, recurrent infections, and persistent colonization. Clinical variables should include inpatient or outpatient status, urinary symptoms, catheterization, pregnancy, comorbidities, previous antimicrobial exposure, recent hospitalization, recurrent UTI, referral source, and community- versus healthcare-associated acquisition. The inclusion of these variables would permit more reliable differentiation of symptomatic infection from asymptomatic bacteriuria or colonization. It would also support adjusted analyses of factors independently associated with fosfomycin resistance. Future laboratory databases should document organism-identification methods, ESBL screening and confirmation procedures, antimicrobial-susceptibility testing platforms, fosfomycin disk and glucose-6-phosphate specifications, MIC methodology, and the CLSI or EUCAST edition used for interpretation. Changes in breakpoints, instruments, standard operating procedures, or laboratory reporting rules should be recorded prospectively.

Raw inhibition-zone diameters and MIC values should be retained wherever feasible. Their availability would allow retrospective application of harmonized breakpoints and more reliable comparison across surveillance periods. Subsequent molecular studies should include larger isolate collections selected using a predefined and temporally structured sampling strategy. Molecular analyses should incorporate resistance-gene carriage testing, sequencing of glpT, uhpT, and murA, ESBL gene subtyping, plasmid characterization, gene copy-number analysis, and whole-genome sequencing where resources permit.

Functional experiments are also required to determine whether the observed transcriptional differences contribute directly to resistance. These may include intracellular fosfomycin uptake assays, transporter-function studies, MurA activity measurements, gene-knockout or complementation experiments, and assessment of fosfomycin-modifying enzyme activity. The transcriptional findings reported here should be independently validated in larger isolate panels before glpT, uhpT, fosA, fosA3, or blaCTX-M are considered mechanistic indicators or candidate biomarkers of fosfomycin resistance.

Finally, multicenter surveillance involving public- and private-sector laboratories, rural and urban populations, and different levels of healthcare across Pakistan would improve external validity. A coordinated national framework integrating standardized laboratory methods, clinical metadata, patient-level deduplication, and periodic molecular characterization would provide a stronger basis for updating local antibiograms, empirical UTI treatment guidance, and antimicrobial-stewardship policy.

## 5. Conclusions

This retrospective, laboratory-based surveillance study identified a temporal shift in the distribution of urinary isolates processed within the participating diagnostic network. The proportion of non-ESBL *Escherichia coli* declined across the study intervals, whereas laboratory-reported ESBL-associated *E. coli* categories became more prominent during the later years. Fosfomycin resistance did not increase consistently across the complete surveillance period. Instead, it declined during 2017–2020 and subsequently increased markedly during 2021–2025, reaching its highest observed level in the most recent interval. These findings indicate that fosfomycin susceptibility within this laboratory population was not temporally stable and should be reassessed through regular local surveillance rather than assumed to remain unchanged. The exploratory molecular sub-analysis of selected archived *E. coli* and *Klebsiella pneumoniae* isolates identified resistance-associated differences in transcript abundance. Resistant isolates showed lower expression of selected fosfomycin uptake-associated genes and higher expression of selected fosfomycin-modifying and ESBL-associated genes. However, these findings were derived from a small, deliberately selected isolate subset and were not supported by comprehensive gene-carriage testing, mutational analysis, plasmid characterization, whole-genome sequencing, direct fosfomycin uptake measurements, transporter-function assays, protein-level analyses, or clonal investigation. They should therefore be interpreted as preliminary transcriptional associations rather than definitive evidence of causal resistance mechanisms, validated molecular biomarkers, or population-wide molecular behavior. These findings support continued laboratory surveillance of major urinary pathogens, periodic updating of local antibiograms, cautious interpretation of empirical fosfomycin activity, and incorporation of current susceptibility data into antimicrobial-stewardship decisions. The conclusions remain subject to the limitations of retrospective isolate-level analysis, incomplete patient-level deduplication, limited clinical metadata, possible historical changes in organism-identification and susceptibility-testing procedures, incomplete reconstruction of year-specific interpretive criteria, and restricted generalizability beyond the diagnostic network studied.

## Data Availability

The data supporting the findings of this study are available from the corresponding author upon reasonable request and subject to institutional and ethical restrictions.
